# Vertical sleeve gastrectomy and semaglutide have distinct effects on skeletal health and heart function in obese male mice

**DOI:** 10.1152/ajpendo.00521.2024

**Published:** 2025-03-12

**Authors:** Caroline de Carvalho Picoli, Sergey Tsibulnikov, Mavy Ho, Victoria DeMambro, Tiange Feng, May Eltahir, Phuong T. Le, Carolyn Chlebek, Clifford J. Rosen, Sergey Ryzhov, Ziru Li

**Affiliations:** Center for Molecular Medicine, MaineHealth Institute for Research, Scarborough, Maine, United States

**Keywords:** bone loss, heart function, semaglutide, vertical sleeve gastrectomy, weight loss

## Abstract

Obesity is a global health challenge associated with significant metabolic and cardiovascular risks. Bariatric surgery and glucagon-like peptide-1 (GLP-1) receptor agonists (GLP-1RAs) are effective interventions for weight loss and metabolic improvement, yet their comparative effects on systemic metabolism—particularly energy metabolism, bone health, and heart function—remain unclear. In this study, obese male mice underwent vertical sleeve gastrectomy (VSG), 6 wk of GLP-1RA (semaglutide) treatment, or sham procedure with saline injection as controls. Dynamic changes in body weight, food intake, fat mass, lean mass, and bone mineral density were monitored. Energy metabolism was assessed using indirect calorimetry. Bone parameters and heart function were evaluated by microcomputed tomography or echocardiography, respectively. Compared with obese controls, VSG and semaglutide treatment comparably reduced body weight and improved glucose metabolism. However, VSG decreased energy expenditure, whereas both treatments similarly promoted lipid utilization. Semaglutide treatment increased ambulatory activity during nighttime. VSG led to significant bone loss, although 6 wk of semaglutide treatment had no significant effects on the skeleton. Cardiovascular outcomes also differed: VSG increased stroke volume without altering heart mass, whereas semaglutide reduced heart mass and transiently elevated heart rate. These findings underscore the importance of carefully weighing the benefits and potential risks of different weight loss treatments when addressing obesity and its systemic complications.

**NEW&NOTEWORTHY:**

Comparative studies of surgical and pharmaceutical approaches to weight loss offer critical insights that can guide clinical decision-making for managing obesity. VSG and semaglutide exhibit comparable efficacy in promoting weight reduction and improving glucose metabolism. VSG reduces energy expenditure, whereas semaglutide increases animal activity during nighttime. VSG leads to significant bone loss, whereas semaglutide preserves bone mass independent of weight loss. VSG improves cardiac outcomes, whereas semaglutide transiently affects heart function.

## INTRODUCTION

Obesity represents a significant public health challenge, with its prevalence sharply increasing worldwide, contributing to numerous comorbidities, including cardiovascular diseases, type 2 diabetes (T2D), and various types of cancers (1–4). The economic burden is substantial; in 2019, the estimated costs of managing overweight and obesity accounted for an average of 2.19% of the global gross domestic product (GDP) across 161 countries (5). If current trends continue, the estimated costs are projected to rise to 3.29% of global GDP by 2060. Therefore, the development of efficient and safe weight-loss strategies has been gaining notoriety.

Bariatric surgery is widely recognized for its effectiveness in managing obesity and T2D (6–10), providing significant improvements in glucose homeostasis, lipid profiles, and insulin sensitivity. One of the mechanisms underlying the benefits of bariatric surgery is the increased secretion of glucagon-like peptide-1 (GLP-1), a gut-derived incretin hormone that enhances insulin release and inhibits glucagon secretion in a glucose-dependent manner (11). The most popular bariatric surgery procedures, such as vertical sleeve gastrectomy (VSG) and Roux-en-Y gastric bypass, significantly boost postprandial GLP-1 levels (12, 13). Thus, the development of GLP-1 receptor agonists (GLP-1RAs), such as semaglutide and liraglutide, has been gaining popularity in managing hyperglycemia and overweight/obesity (13–19).

Beyond the metabolic benefits of VSG and GLP-1RAs, it is worth noting that their side effects are becoming more apparent, especially as more clinical and preclinical data become available. For instance, bariatric surgery has been shown to reduce bone mineral density (BMD) and increase the risk of fractures over time (20–23). VSG-induced bone loss goes beyond calcium and vitamin D deficiency, mechanical unloading, diet preferences, orsex differences (12). Many other factors, such as gut hormones, bile acids, microbiota, and sympathetic drive, may also contribute to VSG-induced bone loss. However, clinical studies with GLP-1RAs have shown inconsistent conclusions on skeleton health showing either beneficial (24), deteriorative (25), or nonsignificant effects (26).

Obesity increases cardiovascular risks (27) and causes adverse cardiac remodeling (28). Bariatric surgery has demonstrated substantial benefits in cardiovascular events; resolving hypertension in 61.7% of patients, and improving hyperlipidemia in 70% of patients (29). With the significant reduction in body weight and cardiac workload, it is not surprising to find that bariatric surgery positively impacts cardiac geometry and function, which is linked to decreased left ventricular (LV) mass, reduced left atrial diameter, and increased LV ejection fraction (LVEF) (30). In a similar scenario, GLP-1RAs generally reduce the risk for major adverse cardiovascular events (31) and improve outcomes in pre-existing heart diseases (32). However, Neves et al. (33) found that GLP-1RA may increase the risk of heart failure in patients with LVEF < 40%. Moreover, long-acting GLP-1RAs, such as liraglutide and albiglutide, have been associated with pronounced increases in heart rate with 6–10 beats/min higher than placebo (34). A mechanistic study in pigs revealed that GLP-1 has direct chronotropic effects on the heart by regulating PKA-dependent phosphorylation of calcium-cycling proteins in the sinus node (35).

Overall, bariatric surgery improves heart function in subjects with pre-existing heart disease but negatively affects bone health, whereas the effects of GLP-1RA treatment remain less clear and warrant further investigation. Comparative studies between these interventions are critical to understanding their benefits and risks. In this study, we directly compared metabolic, skeletal, and cardiac outcomes following VSG, semaglutide treatment, or sham with saline injection. VSG and semaglutide treatment had comparable effects on metabolic improvement. Bone mass was reduced with VSG, but not changed after 6 wk of semaglutide treatment. VSG and semaglutide demonstrated different effects on the heart. VSG increased the left ventricular dimensions, which resulted in the enhanced stroke volume. Semaglutide decreased heart mass while inducing a transient increase in the heart rate.

## MATERIALS AND METHODS

### Vertical Sleeve Gastrectomy

After 8 wk of high-fat diet (HFD; 60% kcal% fat; Research Diets D12492; New Brunswick, NJ) feeding (more details are available in Supplemental Methods), obese male mice underwent either sham or VSG procedures. Under isoflurane anesthesia, a small laparotomy was performed, and 80% of the stomach (along the greater curvature and fundus) was excised in the VSG group using microvessel clips, followed by hand-sewn sutures. Sham surgeries involved applying pressure to the stomach with a microvessel clip for 15 s. Postsurgical care included a single dose of buprenorphine (0.08 mg/kg, sc) and warm saline (1 mL), with daily carprofen (5 mg/kg, sc) injections for 3 days. Mice received a high-energy gel diet (DietGel Boost; ClearH_2_O, Westbrook, ME) 1 day before surgery and 3 days postsurgery, followed by reintroduction of HFD on *day 4*. Body weights were monitored daily for the first week postsurgery and weekly thereafter. Body composition was assessed using dual-energy X-ray absorptiometry (DEXA) at baseline (*day 0*), and *day 21* and *day 42* postsurgery.

### Semaglutide Treatment

A third group of obese male mice received semaglutide (Ozempic; 400 μg/kg sc; Novo Nordisk, Plainsboro, NJ) injections daily for 6 wk (36). The treatment was initiated with a dose escalation (100 μg/kg on *day* 1, 200 μg/kg on *day* 2) before reaching 400 μg/kg for the remainder of the study. Sham mice received daily saline injections (2.5 mL/kg sc) and were used as controls. All injections were administered at 10:00 AM.

### Indirect Calorimetry

A subset of mice from each group was housed in Promethion Metabolic Cage System (Sable Systems, Las Vegas, NV) 4 wk after treatment for 24-h acclimation followed by 72-h data collection. Parameters, such as energy expenditure, respiratory exchange ratio (RER), and ambulatory activity, were monitored as previously described (37). Data acquisition and analysis were performed using Promethion Live (v23.0.08) and Macro Interpreter (v24.5.6) software (Sable Systems International) with detailed scripts for all aspects of data transformation.

### Dual-Energy X-Ray Absorptiometry

DEXA was performed at baseline (*day 0*) and on *day 21* and *day 42* post-treatment using a Hologic Faxitron UltraFocus DXA system (Tucson, AZ). The Faxitron was calibrated with bone and fat phantoms from the manufacturer before each scanning session. Measurements included whole body (excluding the head) bone mineral density (BMD), lean mass, and fat mass, as well as regional BMD for femora, tibiae, lumbar vertebrae (L4), and caudal (tail) vertebra 4.

### Echocardiography

Cardiac function was assessed by echocardiography (ECHO) in vivo on unsedated mice using a Vevo F2 Imaging System (VisualSonics, Inc., Toronto, ON, Canada). M-mode images were obtained from the parasternal short-axis view at the papillary muscles level and analyzed with Vevo LAB 5.8.1. software by another investigator in a blinded manner.

### Microcomputed Tomography Analysis

Tibiae were fixed in 10% formalin for 24 h at 4°C and stored in Sorenson’s buffer. Scans were performed using a high-resolution desktop microtomographic vivaCT 40 system (Scanco Medical AG, Brüttisellen, Switzerland). Scans were acquired using a 10.5 μm^3^ isotropic voxel size, 70 kVp peak X-ray tube intensity, 114 mA X-ray tube current, 250 ms integration time, and were subjected to Gaussian filtration and segmentation. Trabecular bone was analyzed at the metaphysis for bone volume fraction (Tb.BV/TV, %), trabecular thickness (Tb.Th, mm), trabecular number (Tb.N, mm^−1^), trabecular separation (Tb.Sp, mm), and trabecular bone mineral density (Tb.BMD, mg HA/cm^3^). Cortical bone was analyzed at the midshaft for bone area fraction (Ct.Ar/Tt.Ar, %), cortical thickness (Ct.Th, mm), and cortical tissue mineral density (Ct.TMD, mg HA/cm^3^). All analyses were performed using the Scanco software (Medical AG, version 4.05).

### Statistical Analysis

Statistical analyses were performed using GraphPad Prism (version 10). Data normality was determined using the Shapiro–Wilk test. Differences between groups were assessed using either one-way ANOVA or two-way repeated-measures ANOVA with appropriate post hoc tests. One-way ANOVA was used to determine significant differences between independent groups, with a post hoc Tukey test or Dunnett’s test. Two-way repeated-measures ANOVA analysis was used when both time and treatment are considered as factors, evaluating the individual effect of each factor and their interaction. A *P*-value of < 0.05 was considered statistically significant. Data are presented as means ± SD.

To distinguish VSG and semaglutide as independent treatment factors, the statistical analyses for Fig. 2 were performed using R software (version 4.4.1). A two-way ANOVA was applied to evaluate the main effects of therapy (sham, VSG, and semaglutide) and time (day and night) on the dependent variable, as well as their interaction effects. Post hoc pairwise comparisons between therapy groups were conducted using Tukey’s honest significant difference (HSD) test to control for the family-wise error rate associated with multiple comparisons (R code is available in Supplemental Methods). *P* values for each pairwise comparison (sham vs. VSG, sham vs. semaglutide, and VSG vs. semaglutide) were extracted from the Tukey HSD results and reported to indicate statistical significance.

To determine the effects of the variation in lean mass between treatment groups on indirect caloric measurements, including total energy expenditure (EE), resting EE (REE), active EE (AEE), oxygen consumption (VO_2_) and carbon dioxide production (VCO_2_), analysis of covariance (ANCOVA) was performed using JMP statistical software. Lean mass was found to be a significant covariate (*P* < 0.05) with all energy expenditure parameters. Data reported here are shown as per mouse and adjusted means when this covariation was accounted for (38).

## RESULTS

### VSG and Semaglutide Have Comparable Effects on Weight Reduction and Glucose Improvement

**Figure 1.**
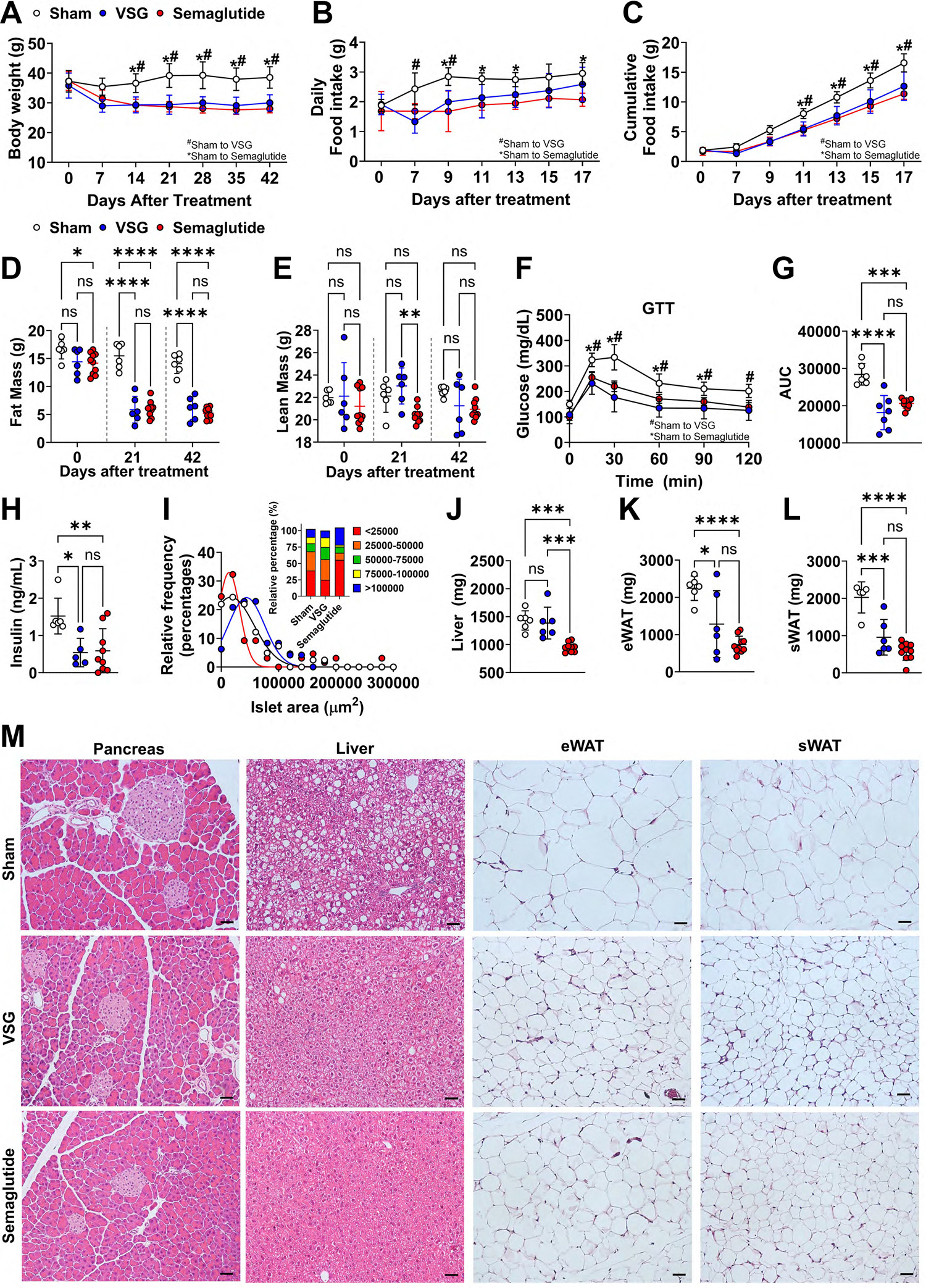
Semaglutide and VSG reduce body weight and improve glucose metabolism in diet-induced obese (DIO) mice. Male C57BL/6J mice were fed an HFD starting from 6 wk old and lasted for 8 wk before vertical sleeve gastrectomy (VSG) or semaglutide treatment. Mice were euthanized 6 wk after treatments. Body weight was monitored weekly (*A*); daily food intake (*B*) and cumulative food intake (*C*) were measured every 2 days from *day 0* to *day 17*. Fat mass (*D*) and lean mass (*E*) were measured by DEXA at *days 0*, *21,* and *42*. *F*: intraperitoneal glucose tolerance test (IPGTT; Supplemental Methods) was performed at *week 4* after treatments. *G*: area under the curve (AUC) was calculated. *H*: circulating insulin concentrations were measured by ELISA. *I*: pancreatic islet sizes were quantified by ImageJ and relative frequency of size distribution was calculated by GraphPad Prism. Soft tissue, including liver (*J*), eWAT (*K*), and sWAT (*L*), weights were measured at the end of study. *M*: representative images of pancreas, liver, eWAT, and sWAT were taken under light microscope at ×20 following paraffin section and H&E staining (Supplemental Methods). Scale bar: 50 lm. Data are expressed as means ± SD. * or #*P* < 0.05; **P < 0.01, ***P < 0.001, and ****P < 0.0001 with a two-way ANOVA (*A*–*F*) and one-way ANOVA test (*G*, *H,* and *J*–*L*). DEXA, dual-energy X-ray absorptiometry; eWAT, epididymal white adipose tissue; H&E, hematoxylin-eosin; sWAT, subcutaneous white adipose tissue.

Male C57BL/6J mice were fed with an HFD for 8 wk before undergoing sham, VSG, or semaglutide treatments. Both VSG and semaglutide treatment caused a sharp initial weight loss (~20%) within the first 14 days; this weight reduction persisted and further decreased to 22%–27% by *day 42* (Fig. 1A). This weight reduction was partially attributed to decreased food intake post-treatment (Fig. 1, B and C). Of note, VSG mice had a transient reduction in food intake during postsurgical recovery phase, which normalized after 2 wk, whereas semaglutide treatment consistently inhibited food intake throughout the experiment. This suggests that the sustained weight loss following VSG may extend beyond mere food intake restriction. DEXA scanning revealed a significant reduction in fat mass with both treatments: 60% and 58% reductions by *day 21* for VSG and semaglutide, respectively, and 58% and 63% by *day 42* (Fig. 1D). Although VSG maintained lean mass, semaglutide showed a trend toward reduced lean mass compared with the sham group (*P* = 0.08 at *day 21* and *P* = 0.11 at *day 42*) (Fig. 1E). Consistent with prior studies (39–42), both VSG and semaglutide greatly improved glucose tolerance (Fig. 1, F and G) and reduced circulating insulin concentrations to a similar extent (Fig. 1H). Histological analysis of pancreatic islets indicated no significant differences between sham and VSG mice, whereas semaglutide-treated mice had a higher proportion (55%) of smaller islets (<25,000 μm^2^), indicating different mechanisms for glucose metabolic improvement between the two treatments (Fig. 1, I and M). Compared with sham mice, lipid deposition in the liver was markedly alleviated following VSG and semaglutide treatment, with semaglutide resulting in significantly smaller liver sizes (Fig. 1, J and M). Both treatments greatly decreased epididymal white adipose tissue (eWAT) and subcutaneous white adipose tissue (sWAT) weights and adipocyte size in each depot (Fig. 1, K–M). These results confirm that both VSG and semaglutide treatments effectively reduced body weight, primarily through fat mass reduction, and improved glucose metabolism, albeit via potentially different mechanisms.

**Figure 2.**
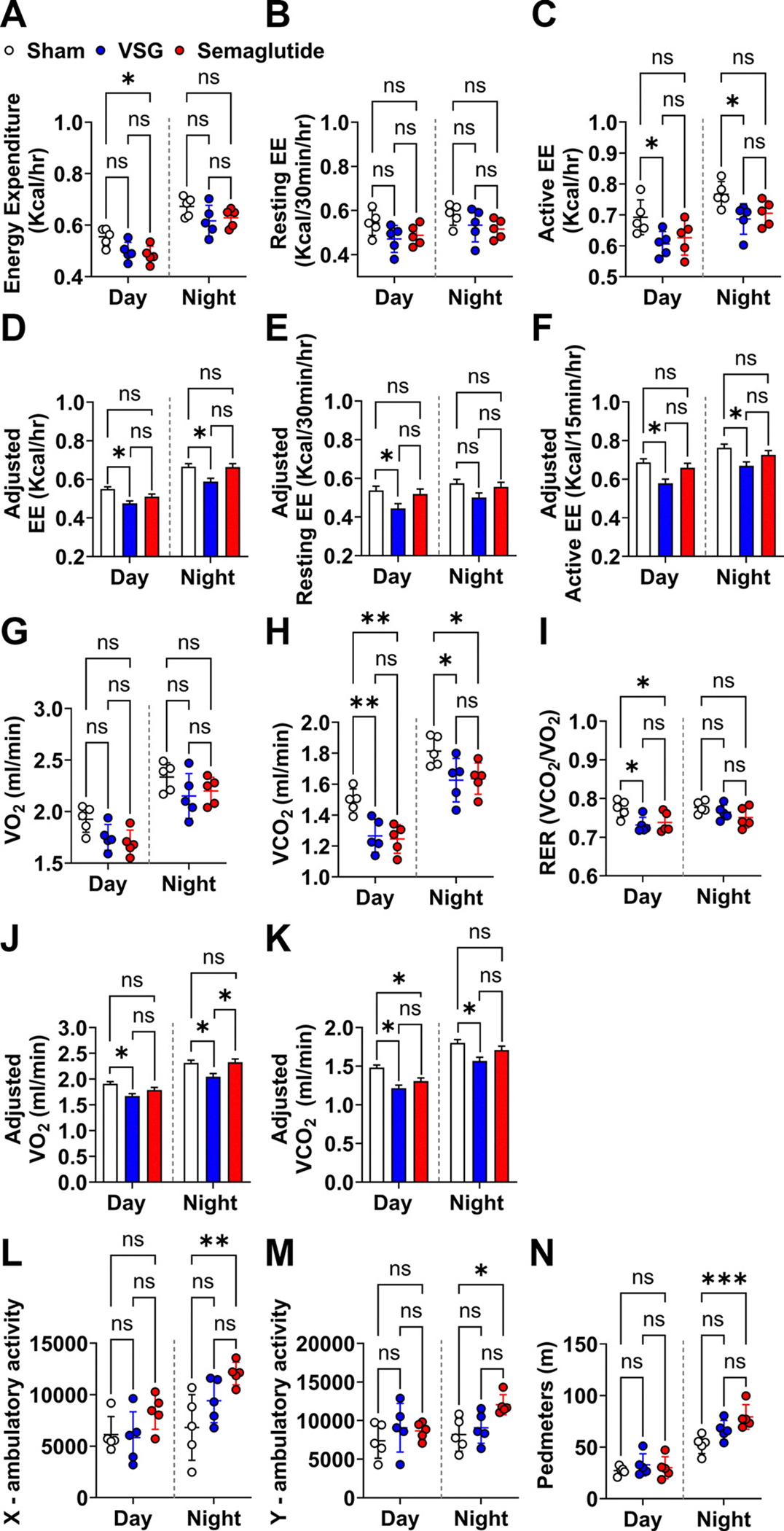
Metabolic cages. A subgroup of mice from each treatment was placed into metabolic cages at 5 wk after treatment for 72 h. Total energy expenditure (EE; *A*), resting EE (*B*), and active EE (*C*) were measured and adjusted to lean mass, which are shown as adjusted EE (*D*), adjusted resting EE (*E*), and adjusted active EE (*F*). Oxygen consumption ($\dot{V}O_{2};G$, CO_2_ production ($\dot{V}{CO}_{2};H$), and respiratory exchange ratio (RER) ($\dot{V}{CO}_{2}/\dot{V}O_{2}$; *l*) were measured, and $\dot{V}O_{2}$ and $\dot{V}{CO}_{2}$ were adjusted to lean mass, which are shown as adjusted $\dot{V}O_{2}$ (*J*) and adjusted $\dot{V}{CO}_{2}$ (*K*). X-ambulatory activity (*L*), Y-ambulatory activity (*M*), and pedestrian locomotion (PedMeters; *N*) were determined. Data are expressed as means ± SD. *P < 0.05; **P < 0.01; and ***P < 0.001 with two-way ANOVA.

To investigate metabolic changes induced by VSG and semaglutide treatments, a subgroup of mice was analyzed in metabolic cages. No statistical differences in total energy expenditure (EE) were identified during the day or night cycles, except for a reduction in daytime EE observed with semaglutide treatment (Fig. 2A). Neither VSG nor semaglutide caused significant changes in resting EE (Fig. 2B). Interestingly, VSG significantly reduced the active EE, and semaglutide treatment showed a similar trend without reaching statistical significance (Fig. 2C). However, when analyzing the overall effects across daytime and nighttime (Supplemental Table S1), both VSG and semaglutide treatments significantly reduced total EE and active EE, with no difference between the two treatments (Supplemental Table S1). Of note, since semaglutide treatment reduced lean mass at *weeks 3* and *4*—when metabolic cage analysis was conducted—it is essential to normalize EE to lean mass. VSG consistently decreased total EE in both day and night cycles when the covariation between lean mass and EE parameters was taken into account via ANCOVA (Fig. 2D). Interestingly, no significant effects of semaglutide treatment on total EE were noted. Analysis of resting EE and active EE revealed similar patterns of significance (Fig. 2, E and F). No significant changes in oxygen consumption (${\dot{VO}}_{2}$) were detected, whereas carbon dioxide production ($\dot{V}{CO}_{2}$) decreased significantly with both treatments during the daytime and nighttime (Fig. 2, G and H). Compared with the sham group, the respiratory exchange ratio (RER) also dropped significantly during the daytime for both treatment groups, indicating a shift toward higher lipid utilization for energy production (Fig. 2I). When the effects across daytime and nighttime were combined, both treatments significantly influenced $\dot{V}O_{2}$, $\dot{V}{CO}_{2}$, and RER compared with the sham group; however, no differences were observed between VSG and semaglutide treatments (Supplemental Table S1). Intriguingly, normalizing $\dot{V}O_{2}$ and $\dot{V}{cO}_{2}$ to lean mass revealed significant reductions only in the VSG group, whereas semaglutide treatment preserved these values at levels comparable to the sham group, except lower $\dot{V}{cO}_{2}$ at daytime (Fig. 2, J and K). In terms of activity, only semaglutide-treated mice exhibited increased *x*-ambulatory, *y*-ambulatory activity, and pedestrian locomotion (PedMeters) during the night cycle (Fig. 2, L–N). Together, our results suggest that VSG and semaglutide treatments exert differential effects on energy metabolism, especially when lean mass is considered as a covariation, and in their impact on activity patterns. Understanding how these weight-loss strategies influence energy balance and physical activity could have implications for optimizing long-term efficacy and sustainability of these interventions.

### VSG Causes Bone Loss, While Semaglutide Treatment Maintains Bone Mass

**Figure 3.**
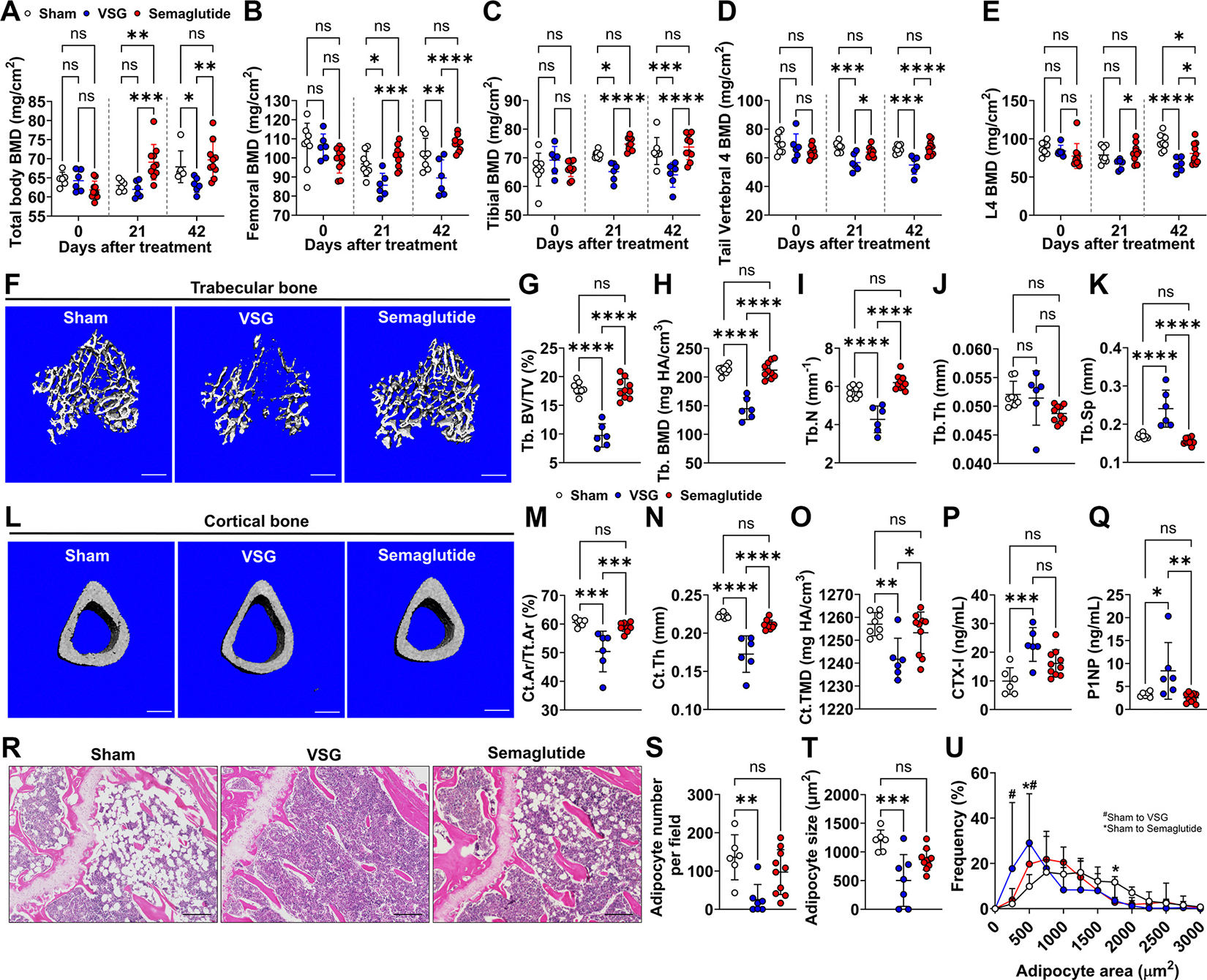
VSG reduces bone mass while semaglutide preserves the bone. Bone mineral density (BMD) for total body (*A*), femur (*B*), tibia (*C*), tail vertebral 4 (*D*), and lumbar vertebral 4 (L4; *E*) were measured by DEXA at baseline (*day 0*), *day 21*, and *day 42* after treatments. *F*: representative 3-D images of trabecular bone in proximal tibiae. Scale bar: 500 μm. Trabecular bone volume fraction (Tb.BV/TV; *G*), BMD (*H*), number (Tb.N; *I*), thickness (Tb.Th; *J*), and separation (Tb.Sp; *K*) were measured by lCT. *L*: representative 3-D images of cortical bone in midshaft tibiae. Scale bar: 500 μm. Cortical bone area fraction (Ct.Ar/Tt.Ar; *M*), thickness (Ct.Th; *N*), and tissue mineral density (Ct.TMD; *O*) were determined by μCT. Circulating CTX-I (*P*) and P1NP (*Q*) were measured by ELISA assay (Supplemental Methods). *R*: representative images of tibiae were taken under light microscope at ×10 following paraffin section and H&E staining. Scale bar: 200 μm. Adipocyte number per field (*S*), average adipocyte size (μm^2^; *T*), and adipocyte size frequency distribution (%; *U*) were quantified. Data are expressed as means ± SD. * or #*P* < 0.05, **P < 0.01, ***P < 0.001, and ****P < 0.0001 with a two-way ANOVA (*A*–*E*, and *U*) or one-way ANOVA test (*G*–*K*, *M*–*Q*, *S,* and *T)*. 3-D, three-dimensional; DEXA, dual-energy X-ray absorptiometry; H&E, hematoxylin-eosin; μCT, microcomputed tomography; VSG, vertical sleeve gastrectomy.

Consistent with previous studies demonstrating bone loss following VSG in humans (22) and rodents (12), our VSG group exhibited a significant reduction in total body BMD 42 days postsurgery, as determined by DEXA scanning (Fig. 3A). Site-specific analysis revealed significant decreases in femoral, tibial, tail vertebral, and lumbar vertebral (L4) BMD starting from *day 21* (Fig. 3, B–E). Despite the striking weight loss following semaglutide treatment, no significant effects on total body or site-specific BMD were observed throughout the 6-wk treatment period (Fig. 3, A–E) except for L4 BMD.

Aligning with the observations determined by DEXA, microCT analysis of mouse tibiae showed significant reductions in trabecular bone volume fraction (Tb.BV/TV) and BMD in VSG mice compared with sham or semaglutide-treated mice (Fig. 3, F–H). The trabecular bone reduction was reflected by the dramatic changes in the microstructure, including decreased trabecular bone number (Tb.N) and increased separation between trabeculae (Tb.Sp) following VSG, without significant changes in trabecular bone thickness (Tb.Th) (Fig. 3, I–K). Similar reductions in cortical bone were determined by significant lower cortical bone area fraction (Ct.Ar/Tt.Ar), cortical thickness (Ct.Th), and total mineral density (Ct.TMD) in VSG mice compared with sham or semaglutide-treated mice (Fig. 3, L–O). Of note, none of the parameters in trabecular and cortical bone morphology was changed by 6 wk of semaglutide treatment, compared with sham mice. Further analysis revealed increased levels of bone turnover markers in VSG mice compared with sham, including both the bone resorption marker C-terminal telopeptide of type I collagen (CTX-1) and the bone formation marker procollagen type I N-terminal propeptide (P1NP). This suggests that VSG induces a state of high bone turnover, potentially contributing to the observed bone loss. In contrast, compared with sham mice, semaglutide-treated mice showed no significant changes in either marker, suggesting stable bone turnover and preservation of bone mass. Understanding these differences could guide clinical decision-making, particularly for patients at risk of osteoporosis or fractures.

Bone marrow adipose tissue (BMAT), a significant component of the bone marrow niche, can either positively or negatively influence bone homeostasis, depending on the metabolic conditions (43). Our previous study found that VSG induces both BMAT depletion and bone loss in parallel (12). Consistent with this notion, VSG depleted the majority of proximal tibial BMAT, with a marked decrease in both the number and size of bone marrow adipocytes (BMAds) (Fig. 3, R–T). Semaglutide treatment showed a trend towards BMAd reduction, but the effect was not statistically significant. Size distribution analysis revealed a clear shift towards smaller BMAds (250 and 500 μm^2^) following VSG, whereas semaglutide treatment increased the proportion of small BMAds at 500 μm^2^ while reducing the prevalence of larger BMAds at 1,750 μm^2^ compared with sham mice (Fig. 3U). Overall, semaglutide appeared to cause less BMAT loss than VSG. This observation is intriguing, as BMAT is well-known to expand upon caloric restriction (44). However, this was not the case with semaglutide treatment, despite its marked reduction in food intake (Fig. 1, B and C). This finding suggests that semaglutide’s effects on the bone marrow niche extend beyond those of food restriction alone.

### VSG and Semaglutide Have Different Effects on Cardiac Function

**Figure 4.**
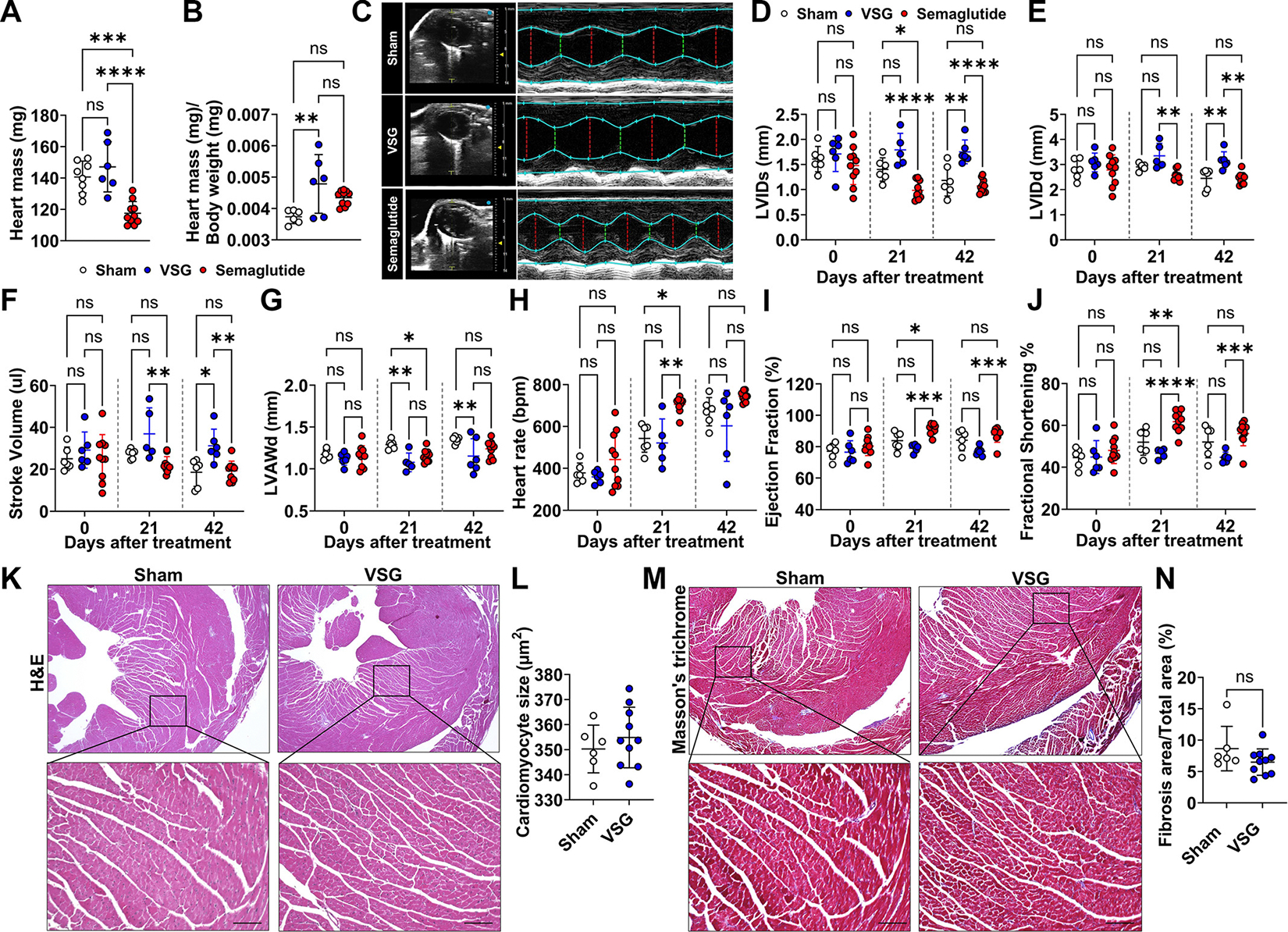
VSG improves heart function while semaglutide has a transient effect. *A*: heart mass (mg) was determined by the end of study. *B*: ratio of heart mass (mg)/body weight (mg) was calculated. *C*: representative echocardiography (ECHO) images of the left ventricle (LV) display dimensions of the ventricular walls, LV cavity, and cardiac function measurements. LV internal diameters in end-systole (LVIDs; *D*) and end-diastole (LVIDd; *E*), stroke volume (μL; *F*), LV anterior wall in diastole (LVAWd; *G*), heart rate (beats/min; *H*), ejection fraction (%; *I*), and fractional shortening (%; *J*) were determined. *K*–*N*: another independent cohort with only sham and VSG mice was used to perform the histological analysis. *K* and *L*: H&E staining was used to quantify cardiomyocyte size. *M* and *N*: Masson’s trichrome staining was used to highlight collagen fibers and quantify the relative proportion of fibrosis area/total area (%). Scale bar: 100 μm. Data are expressed as means ± SD. *P < 0.05, **P < 0.01, ***P < 0.001, and ****P < 0.0001 with a one-way ANOVA (*A* and *B*), two-way ANOVA test (*D*–*J*), or Student’s *t* test (*L* and *N*). H&E, hematoxylin-eosin; VSG, vertical sleeve gastrectomy.

Since heart size correlates with the body size (45, 46), it is not a surprise to see that semaglutide-treated mice, with reduced body mass, had smaller hearts compared with control mice (Fig. 4A). Interestingly, this correlation was absent following VSG because the heart mass in VSG-treated mice remained similar to control, leading to a significantly higher heart-to-body weight ratio in this group (Fig. 4B). Consistent with this notion, echocardiography (ECHO) exam showed increases in left ventricular internal diameter in systole (LVIDs) and diastole (LVIDd) in VSG group compared with groups of sham and semaglutide-treated mice (Fig. 4, D and E). In addition, the stroke volume was significantly elevated in the VSG group by *day 42*, whereas semaglutide-treated mice displayed levels comparable to sham controls (Fig. 4F). To examine if increased LV dimensions are associated with the hypertrophic remodeling, we collected hearts from another independent cohort after confirming the consistent changes in heart function by ECHO. No differences were found in cardiomyocyte size (Fig. 4, K and L), and Mason’s trichrome staining revealed no fibrosis in VSG-treated hearts (Fig. 4, M and N). These results indicate that larger and stronger hearts observed in VSG mice likely reflect physiological, not pathological, adaptations. Of note, both VSG and semaglutide reduced left ventricular anterior wall thickness in diastole (LVAWd) compared with sham mice starting at *day 21*, indicating improvements in both treatment groups (Fig. 4G). In contrast to VSG, semaglutide temporarily increased heart rate and ejection fraction at *day 21* (Fig. 4, H–J), suggesting potential adaptive changes to the treatment during the early phase, which normalized by *day 42*. Overall, these findings suggest that VSG and semaglutide treatment led to improved heart function and structural adaptations without adverse remodeling, most likely using different molecular mechanisms. Understanding these distinct cardiovascular effects can help tailor weight-loss strategies for patients with pre-existing cardiac conditions.

## DISCUSSION

Using a male DIO mouse model, we conducted a parallel comparison between different weight loss approaches, including VSG and semaglutide treatments, to evaluate their metabolic, skeletal, and cardiac outcomes. Our findings revealed comparable efficacy of VSG and semaglutide in reducing body weight and improving glucose metabolism, with different effects on energy metabolism and animal ambulatory activity. It is worth highlighting the distinct effects of VSG and semaglutide treatments on bone health and heart function.

The reductions in body weight and fat mass observed in both VSG and semaglutide treatments align with previous studies in humans and animal models (10, 12, 13, 17), confirming their efficacy in managing obesity and related metabolic disorders. VSG induces weight loss through mechanisms beyond mechanical restriction, malabsorption, and secretion of satiety gut hormones such as GLP-1 and PYY, with growing evidence supporting the gut microbiome as a significant contributor, such as elevated bile acid signaling and the antimicrobial peptide *Reg3g* expression (47–49). Additional mechanisms such as shifts in food preference and changes in energy expenditure also contribute to weight reduction but in a more complex manner (50). Semaglutide, a GLP-1RA, exerts its effects through GLP-1 signaling, which plays a pivotal role in regulating food intake, glucose homeostasis, and energy metabolism (17). GLP-1 receptors are widely expressed in tissues, such as the pancreas (51–53), gastrointestinal tract (54, 55), kidney (16, 56), brain (57, 58), and adipose tissue (59), supporting the diverse physiological benefits of semaglutide (17). These include promoting insulin production, suppressing glucagon secretion, reducing hepatic glucose out-put, inhibiting lipid accumulation (60, 61), reducing energy intake (62), and offering neuroprotective and cardiovascular benefits (63).

Bariatric surgery decreases total EE, primarily due to reduced active EE, indicating the body’s adaptation to significant reductions in fat mass and fat-free mass (64–66). This metabolic adaptation poses challenges for maintaining long-term weight loss. There is limited information about the effects of GLP-1RA treatment on active EE, although reports on resting EE remain inconclusive, with studies showing increases (67), decreases (68), or no change (69, 70). Our indirect calorimetry studies revealed similar trends of reductions in total EE and active EE with both VSG and semaglutide treatment. However, after normalizing to lean mass, semaglutide treatment has no significant effects on EE. Both treatments decreased the RER compared with sham mice, particularly during the daytime, suggesting a shift toward greater lipid utilization. Of note, semaglutide treatment particularly increased ambulatory activity and pedestrian locomotion during the nighttime, suggesting behavioral changes. These differences may have implications for the sustainability of weight loss and warrant further investigation in long-term studies, particularly as it relates to potential effects on the central nervous system (71–73).

Although the effectiveness of VSG and semaglutide treatment for weight loss and metabolic improvements are high-lighted in both clinical and animal studies, it is worth noting that side effects of each treatment should be fully weighed against the benefits, particularly for individuals with pre-existing health issues. For instance, VSG causes anemia, ulcers, hernias, vomiting, dumping syndrome (74, 75), depression (76, 77), and bone loss, evidenced by reductions in BMD at femoral neck, total hip, and lumbar spine (78). Longitudinal human studies confirmed that bariatric surgery is associated with an increased risk of osteoporotic fracture (79, 80). Mechanistically, bariatric surgery-induced bone loss is linked to elevated bone turnover, as shown by increased levels of bone resorption (CTX-I) and formation (P1NP) markers, consistent with human studies following VSG (81, 82).

GLP-1 receptors are also expressed in bone cells, including bone marrow skeletal stem cells, osteoblasts, and osteoclasts (83, 84). In vitro studies have shown that liraglutide treatment promoted osteoblastogenesis (85) and suppressed osteoclastogenesis (86). In vivo studies of GLP-1RA like liraglutide showed variable results, potentially due to differences in compounds, doses, and duration of treatment, as well as the disease conditions. Ovariectomized mice treated with liraglutide or exenatide demonstrated positive effects on trabecular bone without significant changes in cortical bone (84). Treatment of streptozotocin-induced diabetic mice with liraglutide was inefficient to prevent trabecular or cortical bone loss (87). In human studies, a 52-wk semaglutide regimen did not affect P1NP levels but increased bone resorption marker CTx, leading to significant reductions in lumbar spine and total hip BMD (88). These data differ slightly from bariatric surgery, where sustained bone loss is a major concern, suggesting semaglutide treatment may be a safer option for patients at risk of osteoporosis.

Bariatric surgery has protective effects against heart failure, coronary artery disease, myocardial infarction, and cardiovascular mortality (89). These benefits are achieved through several mechanisms. VSG reduces circulating lipids and mitigates hyperglycemia, which in turn alleviates their detrimental effects on the heart. In addition, bariatric surgery decreases systemic inflammation, reduces oxidative stress, and restores endothelial function, resulting in lower risks of atherosclerosis, arrhythmia, and hypertension (90–92). These changes collectively enhance cardiovascular health, as evidenced by improved left ventricular function, reduced cardiac workload, and diminished fat infiltration in the myocardium, ultimately leading to enhanced cardiac performance (93, 94). Our study in male mice confirmed the improvement of heart function, showing an increase in stroke volume and left ventricular dimensions following VSG. Importantly, histological analysis revealed no significant fibrosis or structural damage, suggesting that the cardiac improvements observed with VSG are likely adaptive rather than pathological.

GLP-1RAs application in clinical studies demonstrated overall beneficial effects on heart function. For example, semaglutide has been shown to reduce cardiovascular mortality in patients with pre-existing cardiovascular disease and obesity in the absence of diabetes (32). However, an observational study demonstrated that semaglutide increased the cumulative hazards to congestive heart failure when compared with bariatric surgery (94). Our study highlighted transient increases in heart rate and ejection fraction with semaglutide, which may reflect compensatory mechanisms or stress on the heart. However, the potential long-term cardiac implications of semaglutide treatment remain unclear and warrant further investigation. This is particularly relevant in clinical settings where dose escalation is often used, raising the need for further research to clarify the safety and efficacy of prolonged treatment.

Although both VSG and semaglutide provide significant cardiovascular benefits, bariatric surgery appears to offer more robust and sustained improvements in cardiac function and structure in patients with pre-existing cardiac disease. Semaglutide, though effective for cardiovascular risk reduction in high-risk populations, requires further evaluation to clarify its long-term effects and optimal use. Our study demonstrated that VSG and semaglutide mediated their cardioprotective effects, most likely via independent molecular mechanisms as the pattern and dynamics of those changes were significantly different. Personalized treatment strategies based on patient-specific risk factors and goals remain essential for maximizing cardiovascular outcomes.

In summary, both VSG and semaglutide treatment provide significant benefits for weight loss and metabolic health, but their distinct effects on bone health and cardiac function highlight the importance of tailoring weight-loss strategies based on individual health profiles. The negative effects of VSG on bone morphology and turnover were not observed with semaglutide treatment, potentially making GLP-1RAs a preferable option for populations with pre-existing skeletal fragilities. The improvements in cardiac performance of VSG mice might be more beneficial for patients with pre-existent heart conditions. Comparative studies in clinical populations will be essential to refine our understanding of these treatments and guide their application in obesity and metabolic disease management.

## LIMITATIONS

There are some limitations to this study, despite the valuable insights provided into the comparative effects of VSG and semaglutide treatment. This work is primarily descriptive, lacking deeper mechanistic exploration, which restricts a comprehensive understanding of the pathways underlying the observed outcomes. The mouse model, while informative, may not fully replicate human physiology, necessitating future studies to confirm these findings in human populations. This includes exploring the molecular mechanisms driving the observed differences and evaluating the prolonged safety and efficacy of VSG and semaglutide in clinical settings. Our study characterized overall metabolic changes and functional parameters related to bone and heart health, but future investigations should expand to include additional tissues and organs, such as the pancreas, liver, and brain. Moreover, a more detailed analysis of hormonal and microbiota regulation could yield valuable insights into how these treatments can be optimized and tailored to individual patient needs. We only included male mice in the current study, future studies using female mice are necessary to determine potential sexual dimorphism in response to semaglutide treatment, as women lose more weight in response to semaglutide (95). In addition, the influence of sex on tissue weight loss, such as the proportion of fat versus lean mass, as well as cardiac function, needs further investigation, including comparisons between rodents and humans.

## Supplementary Material

Supplemental Methods: https://doi.org/10.6084/m9.figshare.28365230 and Supplemental Table S1: https://doi.org/10.6084/m9.figshare.28365251.

## Data Availability

Data will be made available upon reasonable request.
